# Gene panel diagnostics reveals new pathogenic variants in pulmonary arterial hypertension

**DOI:** 10.1186/s12931-022-01987-x

**Published:** 2022-03-27

**Authors:** Christina A. Eichstaedt, Zoe Saßmannshausen, Memoona Shaukat, Ding Cao, Panagiota Xanthouli, Henning Gall, Natascha Sommer, Hossein-Ardeschir Ghofrani, Hans-Jürgen Seyfarth, Marianne Lerche, Michael Halank, Janina Kleymann, Nicola Benjamin, Satenik Harutyunova, Benjamin Egenlauf, Katrin Milger, Stephan Rosenkranz, Ralf Ewert, Hans Klose, Marius M. Hoeper, Karen M. Olsson, Mareike Lankeit, Tobias J. Lange, Katrin Hinderhofer, Ekkehard Grünig

**Affiliations:** 1 Center for Pulmonary Hypertension, Thoraxklinik Heidelberg gGmbH at Heidelberg University Hospital, Translational Lung Research Center Heidelberg (TLRC), German Center for Lung Research (DZL), Röntgenstraße 1, 69126 Heidelberg, Germany; 2 Laboratory for Molecular Genetic Diagnostics, Institute of Human Genetics, Heidelberg University, Röntgenstraße 1, 69126 Heidelberg, Germany; 3 Department of Pneumology, Medical and Policlinic II, University Hospital of Gießen and Marburg, Universities of Giessen and Marburg Lung Center (UGMLC), German Center for Lung Research (DZL), Gießen, Germany; 4 Department of Medicine, Imperial College, London, UK; 5 Department of Pneumology, Kerckhoff-Klinik, Bad Nauheim, Germany; 6 Department of Pneumology, Medical Clinic II, University Hospital of Leipzig, Leipzig, Germany; 7 Medical Clinic I, University Hospital of Dresden, Dresden, Germany; 8 Department of Internal Medicine V, Ludwig-Maximilian University of Munich; Asklepios Clinic Gauting, Comprehensive Pneumology Centre Munich (CPC), German Center for Lung Research (DZL), Munich, Germany; 9 Department III of Internal Medicine and Cologne Cardiovascular Research Center (CCRC), Cologne University Heart Center, Cologne, Germany; 10 Department of Internal Medicine B-Cardiology, Intensive Care, Pulmonary Medicine and Infectious Diseases, University of Greifswald, Greifswald, Germany; 11 Department of Pneumology, Department of Medicine II, University Medical Center Hamburg-Eppendorf, Hamburg, Germany; 12 Clinic for Pneumology, Hannover Medical School, Hannover and German Centre for Lung Research (DZL), Hannover, Germany; 13 Department of Internal Medicine and Cardiology, Campus Virchow Klinikum (CVK), Charité - University Medicine Berlin, Berlin, Germany; 14 Center for Thrombosis and Hemostasis, University Medical Center Mainz, Mainz, Germany; 15 Department of Internal Medicine II, Division of Pneumology, University Medical Center Regensburg, Regensburg, Germany

**Keywords:** Pulmonary arterial hypertension, Genetic testing, Bi-allelic variants, Gene panel diagnostics

## Abstract

**Background:**

A genetic predisposition can lead to the rare disease pulmonary arterial hypertension (PAH). Most mutations have been identified in the gene *BMPR2* in heritable PAH. However, as of today 15 further PAH genes have been described. The exact prevalence across these genes particularly in other PAH forms remains uncertain. We present the distribution of mutations across PAH genes identified at the largest German referral centre for genetic diagnostics in PAH over a course of > 3 years.

**Methods:**

Our PAH-specific gene diagnostics panel was used to sequence 325 consecutive PAH patients from March 2017 to October 2020. For the first year the panel contained thirteen PAH genes: *ACVRL1, BMPR1B, BMPR2, CAV1, EIF2AK4, ENG, GDF2, KCNA5, KCNK3, KLF2, SMAD4, SMAD9* and *TBX4.* These were extended by the three genes *ATP13A3, AQP1* and *SOX17* from March 2018 onwards following the genes’ discovery.

**Results:**

A total of 79 mutations were identified in 74 patients (23%). Of the variants 51 (65%) were located in the gene *BMPR2* while the other 28 variants were found in ten further PAH genes. We identified disease-causing variants in the genes *AQP1, KCNK3* and *SOX17* in families with at least two PAH patients*.* Mutations were not only detected in patients with heritable and idiopathic but also with associated PAH.

**Conclusions:**

Genetic defects were identified in 23% of the patients in a total of 11 PAH genes. This illustrates the benefit of the specific gene panel containing all known PAH genes.

**Supplementary Information:**

The online version contains supplementary material available at 10.1186/s12931-022-01987-x.

## Background

Pulmonary arterial hypertension (PAH) is a rare disease characterised by a remodelling, narrowing or even occlusion of the small pulmonary vessels. This leads to an increased pulmonary vascular resistance, right heart failure and death [1]. The abnormal proliferation of mainly the small pulmonary arteries and rarely the venules in PAH patients can be induced by pathogenic variants (mutations) in genes involved in the transforming growth factor beta (TGF-β) pathway. In a large family with heritable PAH (HPAH) we identified linkage of the disease to chromosome 2q31-32 [2]. In this region the bone morphogenetic protein receptor 2 (*BMPR2*) gene has been discovered and the first mutations have been detected in families with HPAH [3, 4]. Since the year 2000, more than 650 different heterozygous pathogenic variants have been identified in this PAH gene [5]. It has been shown to be the most affected gene in HPAH (76–86%) and non-familial idiopathic PAH (12–14%) [6, 7]. Since the discovery of *BMPR2* as a causative PAH gene, genetic defects have been detected in at least 15 further genes, most of them also involved in the BMPR-II signalling pathway [8, 9]. The co-factors of BMPR-II endoglin (*ENG*) and activin receptor like kinase 1 (*ACVRL1*) are predominantly altered in hereditary haemorrhagic telangiectasia associated PAH [10, 11]. Further PAH genes belong to the bone morphogenetic proteins such as BMP9 (*GDF2*), which initiates the BMPR-II signalling cascade or the SMAD proteins such as SMAD8 (*SMAD9*) and SMAD4 (*SMAD4*), which convey BMPR-II signalling from the cell membrane to the nucleus [8]. Over the years, it has become increasingly apparent that patients with distinct forms of PAH were shown to have a higher prevalence of pathogenic variants in specific genes. For example, we detected a mutation in the *KLF2* gene in one family [9]. Patients with heritable pulmonary veno-occlusive disease (PVOD) carry pathogenic variants in the gene *EIF2AK4*, while in patients with congenital heart disease associated PAH and some paediatric cases of PAH mutations were identified in the transcription factors *SOX17* and *TBX4* [7, 12, 13].

Thus, it is crucial in a genetic diagnostic setting to always include also the most recently discovered genes. Initially, only Sanger sequencing of the three first described genes *BMPR2*, *ACVRL1* and *ENG* had been conducted. Due to technical advances a switch to a PAH-specific gene panel based on next generation sequencing has enabled the analysis of all known PAH genes [14]. The adaptable nature of the panel ensures a comprehensive genetic diagnostic testing which may even assist in the correct diagnosis of PAH patients. In 2017 we began using our recently patented PAH gene panel (EP3507380) in the routine diagnostics setting as the largest German referral centre for genetic testing in PAH. To the initial set of 13 genes three additional genes (*AQP1*, *ATP13A3* and *SOX17*) were added subsequent to their discovery as PAH causative genes. Since only a limited number of mutations has been published in the newer PAH genes, it is crucial to describe their role in the genetic background of a representative, unbiased set of consecutive PAH patients. Hence, the aim of this study was to demonstrate the value and the relevance for genetic testing in the clinical routine by assessing the actual proportion and range of genetic predisposition across genes and PAH aetiologies.

## Methods

### Patient cohort

All 325 PAH patients were diagnosed according to current guidelines including right heart catheterisation [1]. Samples from HPAH, idiopathic PAH (IPAH), associated PAH and PVOD patients were sent for genetic diagnostic testing to the Institute of Human Genetics at Heidelberg University between March 2017 and October 2020. Only unrelated PAH patients, who received a complete genetic diagnostic testing, were included in the reported cohort. Further targeted genetic testing was conducted in three HPAH families after the disease-causing familial mutation had been identified. Patients included in this study underwent genetic counselling and signed written informed consent for their data and samples to be used for research purposes. The Ethics Committee at Heidelberg University had no objections against this study (project identification codes 065/2001 and S-426/2017).

### Genetic testing

DNA was extracted from 3 to 10 ml of EDTA-blood samples using an automated procedure (Autopure or QIAsymphony, QIAGEN, Germany). The quantity of DNA was measured with a spectrophotometer (Nanodrop and Qubit, Thermo Fisher Scientific, USA). The extracted DNA was diluted according to the manufacturer’s protocol and prepared with a customised SureSelect QXT kit to enrich for the PAH genes of interest (Agilent, Germany). DNA was sequenced by next-generation sequencing (NGS) using a PAH-specific gene panel including the following PAH diagnostic genes: *ACVRL1, BMPR1B, BMPR2, CAV1, EIF2AK4, ENG, GDF2, KCNA5, KCNK3, KLF2, SMAD4, SMAD9* and *TBX4*. Following their discovery as PAH genes in March 2018 the three genes *ATP13A3, AQP1* and *SOX17* were added to the panel and sequenced in all subsequent patients. The procedure was carried out as previously described [14] and performed on the MiSeq (Illumina, USA). The entire coding region of all PAH genes plus the exon–intron boundaries were analysed. The PAH-specific gene panel received the recognition as European patent in March 2021 (EP3507380).

For a complete genetic testing of PAH patients, multiplex ligation-dependent probe amplification (MLPA) was performed to identify gross deletions and duplications in the genes *ACVRL1, BMPR2* and *ENG* (P093-C2, MRC-Holland, Netherlands)*.* Familial variants were sought by Sanger sequencing in family members (ABI Genetic Analyzer 3130xl, Applied Biosystems, USA). For variants predicted to result in a splice site loss > 2 base pair (bp) away from the canonical splice site, a new EDTA blood was requested. RNA was extracted using standard protocols (RNeasy Mini Kit, Qiagen). Complementary DNA was transcribed with superscript II reverse transcriptase (Life Technologies, USA) with random hexamer primers (Roche Diagnostics Deutschland GmbH, Germany). Polymerase chain reactions were designed with primers located in adjacent exons to the predicted alternative or lost splice site. Sizes of amplified products were measured by agarose gel electrophoresis and Sanger sequenced.

### Variant characterisation

**Fig. 1:**
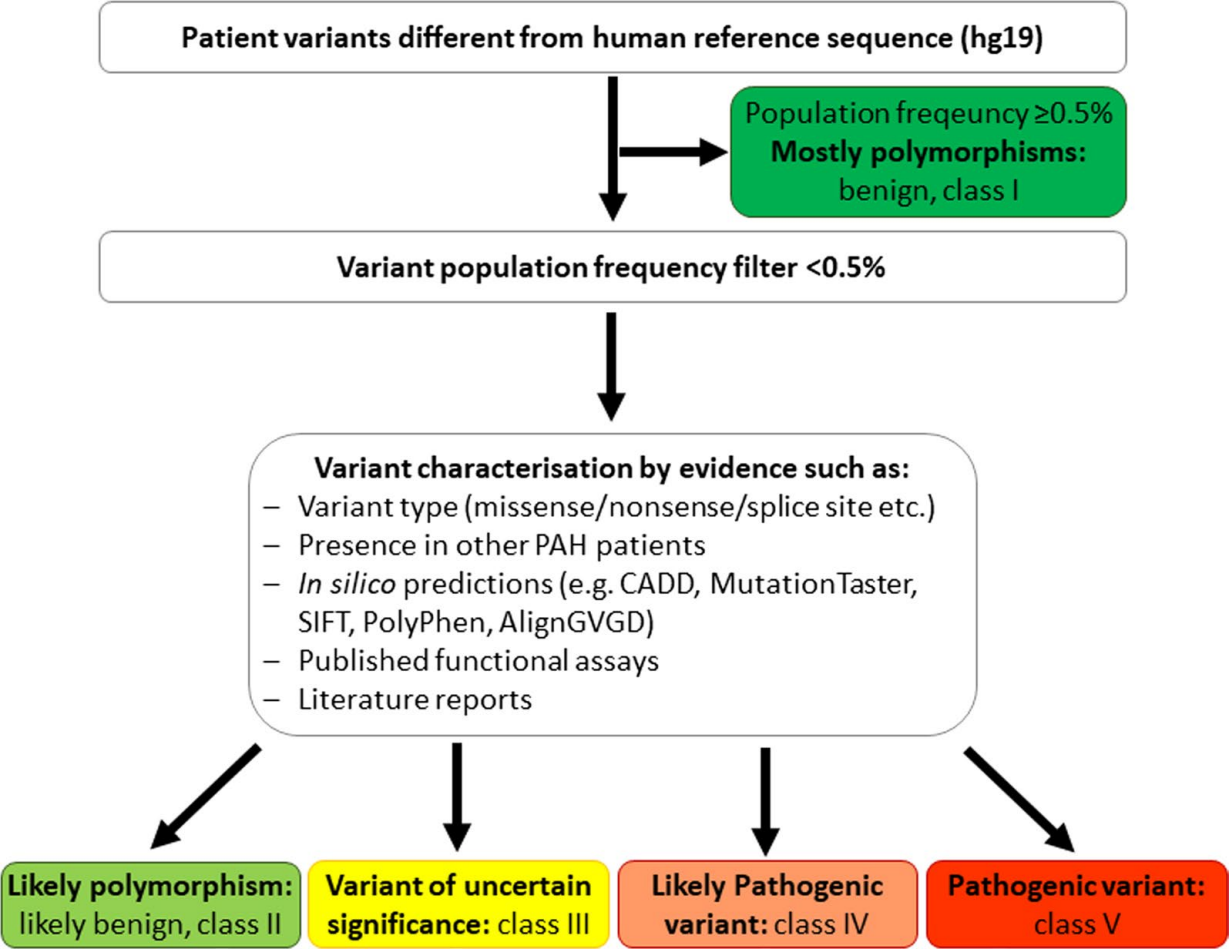
Flow chart of variant characterization process. Variants were firstly characterised by their population frequency in the genome aggregation database (GnomAD). Those with a frequency above 0.5% were excluded from further analysis. Secondly, all available evidence for each variant was taken into consideration to obtain a final classification. Likely pathogenic and pathogenic variants were termed “mutations”

Data quality of each NGS run was confirmed by the amount of generated data, the average base call error rate ≤ 0.01% (≥ Q30) and a cluster density around 1000. The coverage was set to at least 100 reads for each exon and exon–intron boundary. The resulting variants were compared to the human reference genome (GRCh37/hg19). Any variants equal or above 0.5% population frequency in the genome aggregation database (GnomAD) with approximately 130.000 participants were excluded from further analyses. The remaining variants were characterised following the variant interpretation criteria published in the guidelines of the American College of Medical Genetics and Genomics (ACMG) [15]. They were classified as either class V pathogenic variants, class IV likely pathogenic variants, class III variants of uncertain significance or class II likely benign polymorphisms [15, 16]. The variant characterisation process is illustrated in Fig. 1. Pathogenic and likely pathogenic variants were defined for this work as “mutations”. To differentiate between class III, IV and V firstly, in silico prediction programmes (SIFT, Polyphen, AlignGVGD, MutationTaster; embedded in Alamut Visual versions 2.11-2.14) and CADD were employed. The possible impact on splice sites was assessed using SpliceSiteFinder-like, MaxEntScan, NNSPLICE, GeneSplicer and Human Splicing Finder. Finally, the variants were sought in the Human Genome Variation Database (HGMD versions 2017.1-2020.3) and in literature. Evidence was summarised in a medical report returned to the requesting physician to communicate the results to the patient during genetic counselling.

### Statistical analyses

Statistical analyses were performed by a medical statistician (NB). Descriptives were displayed as mean with standard deviation or frequency and respective percentage. Differences between patients with no mutation, *BMPR2* mutation and any other mutation were analysed by analysis of variance (ANOVA) and with post-hoc two-sided t-tests in case of significant global test. P-values ≤ 0.05 were considered as significant. For comparison of categorical variables, chi-square tests were used. All analyses were performed with IBM SPSS V27.0 (Armonk, NY: IBM Corp).

## Results

### Patient cohort

In the cohort of 325 consecutive PAH patients, 70% were female (n = 227) with an age of onset of 47 ± 17 years (Table 1). The majority (62%) were in WHO functional class III at diagnosis, with severely impaired haemodynamics. Most patients (41%) were treated with a double combination therapy at time of this study, almost a third received triple combination therapy including prostacyclin (28%). Vasoresponsiveness was identified in 6%. Of the 325 patients 62% were classified as idiopathic PAH (IPAH) and 15% as heritable PAH (HPAH), (Table 1). Included were also patients with APAH forms (n = 55), PVOD (n = 16), persistent PH of the newborn (n = 3) and drugs and toxins induced PAH.

**Table 1:** Patient characteristics at time of diagnosis stratified by *BMPR2* and other mutations

Parameter	All patients (n = 325)			No mutation (n = 251)			*BMPR2* mutation (n = 50)				Other mutation (n = 24)				Overall p-value^#^
	Mean ± SD or %		n*	Mean ± SD or %		n*	Mean ± SD or %		n*	p-value^+^	Mean ± SD or %		n*	p-value^+^	
Age at diagnosis (years)	47	± 17	325	48	± 18	251	47	± 17	50		40	± 17	24		0.069
Females	227	70%	325	177	71%	251	38	76%	50		12	50%	24		0.066
6 min walking distance (m)	393	± 124	249	390	± 129	195	412	± 96	40		380	± 128	15		0.529
N-terminal pro-brain natriuretic peptide (ng/l)	1539	± 2331	225	1479	± 2428	178	2062	± 1955	34		996	± 1796	13		0.285
Pro-brain natriuretic peptide (pg/ml)	315	± 291	19	320	± 311	14	235	199	4		11	-	1		0.602
Diagnosis			325			251			50	< 0.001			24	< 0.001	< 0.001
IPAH	201	61.8%		173	68.9%		20	40.0%			8	33.3%			
HPAH	47	14.5%		9	3.6%		29	58.0%			9	37.5%			
CHD-APAH	28	8.6%		27	10.8%		0	–			1	4.2%			
CTD-APAH	23	7.1%		22	8.8%		0	–			1	4.2%			
PVOD	16	4.9%		12	4.8%		0	–			4	16.7%			
Persistent PH of the newborn	3	0.9%		3	1.2%		0	–			0	-			
Drugs and toxins induced PAH	3	0.9%		1	0.4%		1	2.0%			1	4.2%			
Portal hypertension	2	0.6%		2	0.8%		0	-			0	–			
HIV-APAH	2	0.6%		2	0.8%		0	-			0	-			
WHO functional class			274			215			42				17		0.880
WHO functional class I	3	1%		3	1%		0	0%			0	0%			
WHO functional class II	82	30%		67	31%		10	24%			5	29%			
WHO functional class III	169	62%		130	61%		29	69%			10	59%			
WHO functional class IV	20	7%		15	7%		3	7%			2	12%			
Current treatment			281			222			42	< 0.001			17	0.017	< 0.001
Mono-therapy	71	25%		67	30.2%		1	2.4%			3	17.7%			
Double combination therapy	114	41%		94	42.3%		15	35.7%			5	29.4%			
Triple combination therapy	80**	28%		45	20.3%		26	61.9%			9	52.9%			
Calcium channel blockers alone	16	6%		16	7.2%		0	-			0	-			
Haemodynamics															
Mean pulmonary artery pressure (mmHg)	49	± 15	274	47	± 15	216	55	± 11	42	0.011	54	± 15	16	0.253	0.006
Pulmonary artery wedge pressure (mmHg)	8.7	± 3.8	257	10.1	± 3.8	200	7.8	± 2.9	41	0.002	7.6	± 3.4	16	0.112	< 0.001
Pulmonary vascular resistance (Wood Units)	10.8	± 6.0	249	9.9	± 5.7	195	15.5	± 5.8	38	< 0.001	10.9	± 4.7	16	1.0	< 0.001
Cardiac output (l/min)	4.3	± 1.7	248	4.5	± 1.7	194	3.4	± 1.1	38	< 0.001	4.2	± 1.7	16	1.0	< 0.001
Cardiac index (l/min/m^2^)	2.4	± 0.8	226	2.5	± 0.8	175	1.9	± 0.5	37	< 0.001	2.4	± 0.7	14	1.0	< 0.001

^+^Post-hoc t-test comparison with “no mutation” group in case of significant ANOVA

^#^ANOVA including all groups

*n varies for each parameter, exact numbers are listed in this column

**One patient received imatinib on compassionate use basis in addition to sildenafil, macitentan and treprostinil

*APAH* associated pulmonary arterial hypertension, *CHD* congenital heart disease, *CTD* connective tissue disease, *HHT* hereditary haemorrhagic telangiectasia, *HIV* human immunodeficiency virus, *HPAH* heritable pulmonary arterial hypertension, *IPAH* idiopathic pulmonary arterial hypertension

### Pathogenic variant overview

**Fig. 2:**
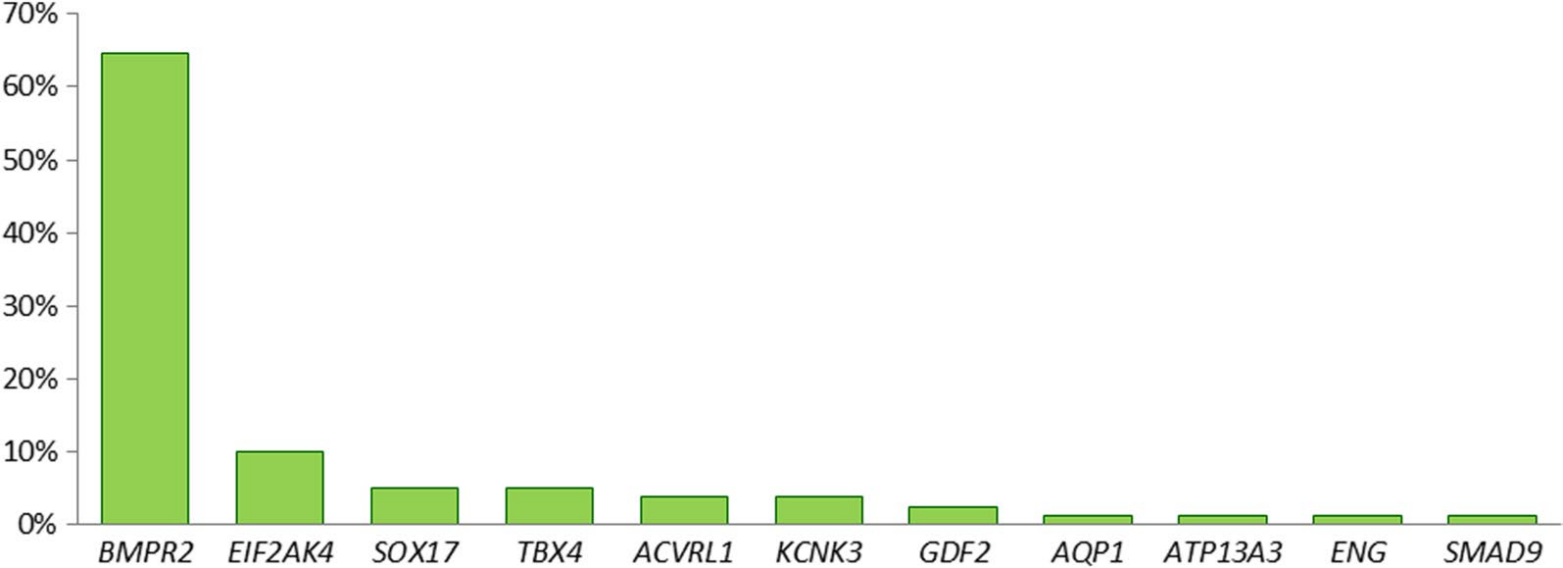
Distribution of mutations across genes. The majority of mutations (65%) was identified in the *BMPR2* gene. The remaining variants were found in 10 further PAH genes. Six large deletions or duplications in the *BMPR2* gene were identified in nine patients using multiplex ligation-dependent amplification (MLPA)

**Fig. 3:**
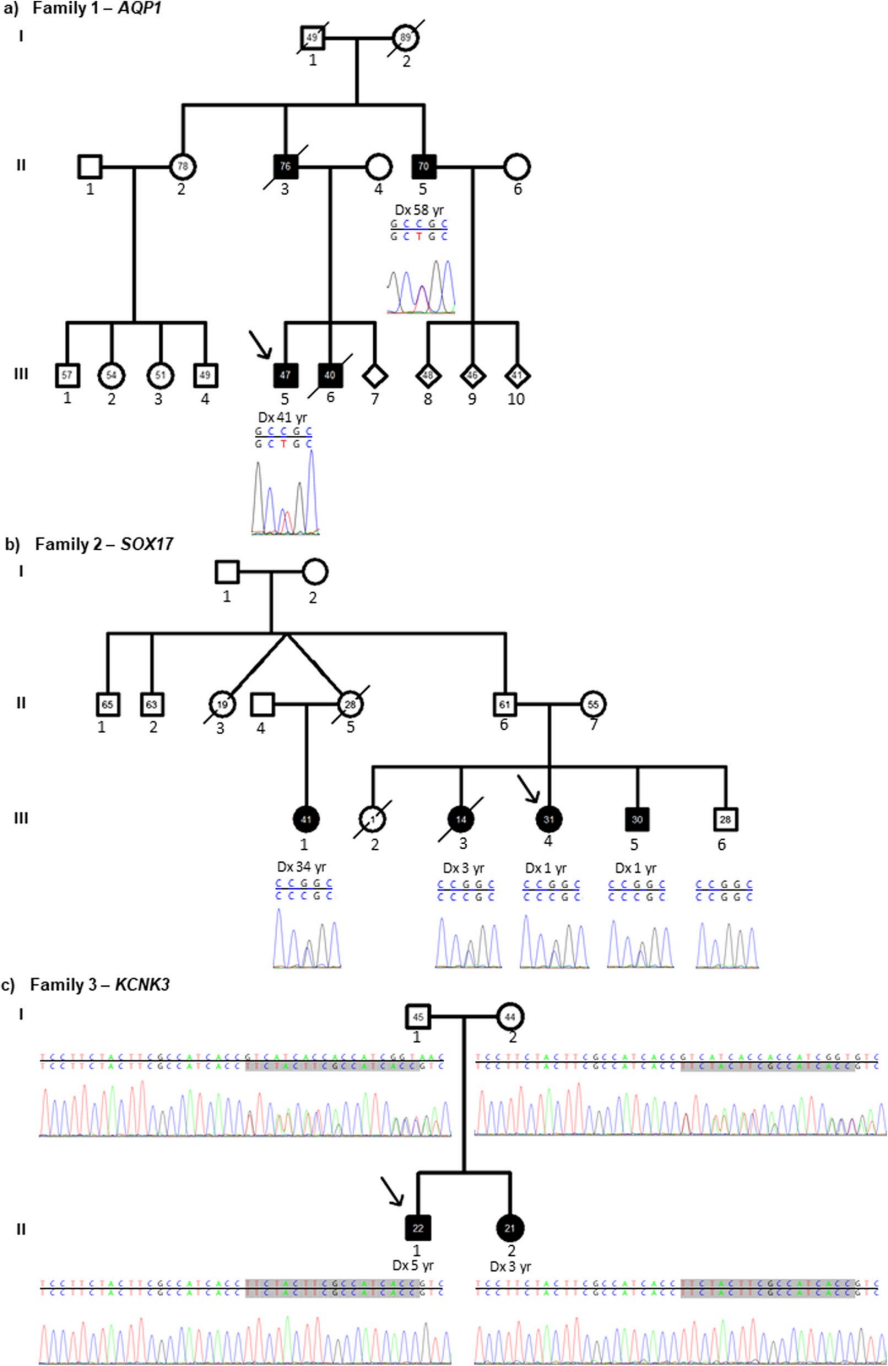
Pedigrees of three non-*BMPR2* families. **a** Pedigree of *AQP1* family**.** The index patient and his uncle carried the heterozygous missense mutation in exon 1 of the gene *AQP1* X1 c.376C > T p.(Arg126Cys). The brother and father of the index patient had died of PAH. No further samples were available from the family. **b** Pedigree of *SOX17* family. The familial heterozygous missense mutation was located in exon 2 of the gene *SOX17* c.413G > C p.(Arg138Pro). It was prevalent in all three siblings and cousin of the index with PAH, while being absent in the only healthy sibling of the index patient. **c** Pedigree of *KCNK3* family. Both parents were heterozygous for the 18 bp duplication in exon 1 c.250_267dup p.(Val84_Thr89dup) displayed in grey. Downstream to the left are the same 18 bp present in the wild-type reference sequence. Both children developed PAH and were homozygous for the duplication. Filled symbol: PAH, empty symbol: healthy family member; the number within the symbols is current age or age at death, Dx: age at diagnosis; upper sequence: wild type reference sequence, bottom sequence: sequence of family member; grey: duplicated sequence not present in the wild-type reference sequence

A total of 79 rare, disease-causing variants (mutations) were identified in 74 of the 325 (23%) patients. The majority of these variants (65%) were located in the gene *BMPR2* but the remaining 28 (35%) variants were found in the genes 10 other genes (Fig. 2, Table 2). Apart from mutations in *BMPR2*, familial PAH patients also carried mutations in the genes *ACVRL1*, *AQP1, GDF2, KCNK3, SMAD9* and *SOX17* (Table 2 and Fig. 3). In total, the disease-causing variant could be identified in 83% of the HPAH patients. In the group of IPAH patients, 13% had mutations in six different genes (Table 2). Two patients with hereditary haemorrhagic telangiectasia and PAH carried mutations in *ACVRL1* and were classified as HPAH. Four patients with PVOD presented with mutations in *EIF2AK4*. Out of three drugs and toxins induced PAH patients two were mutation carriers (66%). In contrast, in the group of APAH patients with connective tissue disease (n = 23) and congenital heart disease APAH (n = 28) only one patient each had a genetic predisposition. The 28 pathogenic variants in non-*BMPR2* genes are listed in Table 3, the 51 pathogenic variants in the gene *BMPR2* are provided in Additional file 1: Table S1.

**Table 2:** Distribution of 79 (likely) pathogenic variants in 74 PAH patients

	IPAH (n = 201)	HPAH (n = 47)	CHD-APAH (n = 28)	CTD-APAH (n = 23)	Drugs and toxins induced PAH (n = 3)	PVOD (n = 16)
*BMPR2*	20	30	–	–	1	–
*ACVRL1*	–	3	–	–	–	–
*AQP1*	–	1	–	–	–	–
*ATP13A3*	–	–	–	–	1	–
*EIF2AK4*	–	–	–	2	–	6
*ENG*	1	–	–	–	–	–
*GDF2*	1	1	–	–	–	–
*KCNK3*	1	2	–	–	–	–
*SMAD9*	–	1	–	–	–	–
*SOX17*	2	1	1	–	–	–
*TBX4*	4	–	–	–	–	–
Total	29 in 27 patients	39 in 39 patients	1 in 1 patient	2 in 1 patient	2 in 2 patients	6 in 4 patients

*APAH* associated pulmonary arterial hypertension, *CHD* congenital heart disease, *CTD* connective tissue disease, *HHT* hereditary haemorrhagic telangiectasia, *HPAH* heritable pulmonary arterial hypertension, *IPAH* idiopathic pulmonary arterial hypertension, *PVOD* pulmonary veno-occlusive disease

**Table 3:** (Likely) pathogenic variants in non-*BMPR2* genes

Gene	Exon	DNA	Protein	CADD score	GnomAD count	Diagnosis
*ACVRL1*	3	c.205T > C	p.(Cys69Arg)	25	0	HPAH (HHT)
*ACVRL1*	5	c.578T > C	p.(Leu193Pro)	31	0	HPAH (HHT)
*ACVRL1*	10	c.1450C > T	p(Arg484Trp)	26	0	HPAH
*AQP1*	1	c.376C > T	p.(Arg126Cys)	26	0	HPAH
*ATP13A3*	14	c.1540C > T	p.(Gln514*)	NA (Nonsense)	0	Drugs and toxins induced PAH [17]
*EIF2AK4*	1	c.1A > T	p.(Met1?)^b^	NA (Start loss)	0	PVOD
*EIF2AK4*	6	c.595-1G > A	intronic ^b^	35	0	PVOD
*EIF2AK4*	6	c.641delA	p.(Lys241Argfs*21)^a^	NA (Deletion)	0	PVOD
*EIF2AK4*	9	c.1362C > A	p.(Cys454*)^b^	NA (Nonsense)	0	CTD-APAH
*EIF2AK4*	21	c.2965C > T	p.(Arg989Trp)^b^	31	5	PVOD
*EIF2AK4*	24	c.3380C > T	p.(Ala1127Val)^b^	26	0	PVOD
*EIF2AK4*	25	c.3443T > C	p.(Leu1148Ser)^b^	31	0	CTD-APAH
*EIF2AK4*	31	c.4260G > A	p.(Trp1420*)^a^	NA (Nonsense)	0	PVOD
*ENG*	12	c.1646G > A	p.(Cys549Tyr)	26	0	IPAH
*GDF2*	2	c.857dupA	p.(Leu287Alafs*11)	NA (Duplication)	0	IPAH
*GDF2*	1	c.329G > A	p.(Arg110Gln)	32	0	HPAH
*KCNK3*	2	c.641T > C	p.(Leu214Pro)	27	0	IPAH
*KCNK3*	2	c.340G > A	p.(Ala114Thr)	26	0	HPAH
*KCNK3*	1	c.250_267dup TTCTACTTCGCCATCACC	p.(Val84_Thr89dup)^a^	NA (Duplication)	0	HPAH
*SMAD9*	2	c.29T > C	p.(Leu10Pro)	27	0	HPAH
*SOX17*	1	c.273_277delAGACC	p.(Asp92Alafs*68)	NA (Deletion)	0	IPAH
*SOX17*	2	c.413G > C	p.(Arg138Pro)	33	0	HPAH
*SOX17*	2	c.1245A > G	p.(*415Trp)	Stop loss	0	CHD-APAH
*SOX17*	2	c.499_520del	p.(Leu167Trpfs*213)	NA (Deletion)	0	IPAH
*TBX4*	2	c.278G > A	p.(Gly93Asp)	33	0	IPAH
*TBX4*	3	c.400delT	p.(Trp134Glyfs*38)	NA (Deletion)	0	IPAH
*TBX4*	6	c.709_710ins56bp	p.(Gln237Profs*18)^b^	NA (Insertion)	0	IPAH
*TBX4*	8	c.1543G > A	p.(Glu515Lys)^b^	24	0	IPAH

^a^Homozygous variant

^b^Together with 2nd variant in same gene

*APAH* associated pulmonary arterial hypertension; *CADD score* Combined Annotation Dependent Depletion score, summation score based on different in silico prediction programmes, *CHD* congenital heart disease, *CTD* connective tissue disease, *GnomAD* Genome Aggregation Consortium Database with n = 125,748 samples, *HHT* hereditary haemorrhagic telangiectasia, *HPAH* heritable pulmonary arterial hypertension, *IPAH* idiopathic pulmonary arterial hypertension, *PVOD* pulmonary veno-occlusive disease. Used Ensembl transcripts and respective reference sequences: *ACVRL1*: ENST00000267008.3, NM_000020; *AQP1*: ENST00000311813.11, NM_198098.3; *ATP13A3*: ENST00000256031.4, NM_024524.3; *EIF2AK4*: ENST00000263791.1 NM_001013703; *ENG:* ENST00000373203.2, NM_001114753; *GDF2*: ENST00000249598, NM_016204.2; *KCNK3*: ENST00000302909, NM_002246.2; *SMAD9*: ENST00000379826.1, NM_001127217; *SOX17*: ENST00000297316, NM_022454.3; *TBX4*: ENST00000240335.1, NM_018488.3

### Clinical characteristics of variant carriers

From the 24 non-*BMPR2* mutation carriers 50% were male, in contrast to the approximately 25% male patients in the *BMPR2*-mutation carrier and no mutation group (Table 1). This difference was not statistically significant likely due to the small sample size in the non-*BMPR2* group. *BMPR2*-mutation carriers showed worse haemodynamic parameters at diagnosis compared to PAH patients without a genetic predisposition. The mean pulmonary artery pressure (p = 0.011) and pulmonary vascular resistance (p < 0.001) were higher while cardiac output and cardiac index were lower (both p < 0.001). Carriers of pathogenic variants in other genes were haemodynamically not statistically different to non-carriers.

Patients with any pathogenic variant more often received a double or triple therapy compared to those patients without a genetic predisposition (p < 0.001). Of the 67 patients receiving mono-therapy, 94% had no pathogenic variant (Table 1). None of the 16 vasoresponders exclusively receiving calcium channel blockers carried a pathogenic variant. The only vasoresponder with a genetic predisposition received triple therapy in addition to a calcium channel blocker.

### HPAH families with mono-allelic pathogenic variants

Pathogenic variants were identified in seven genes in 39 HPAH families (Table 2). Around one third had further family members tested for the familial variant (data not shown). In the following, we focused on those families with at least two PAH patients tested for the familial variant in a non-*BMPR2* gene.

The first family had a heterozygous missense mutation in the gene *AQP1*. The mutation c.376C > T p.(Arg126Cys) led to the exchange of the amino acid arginine by cytosine (Fig. 3A). It was absent in the genome aggregation database and had been previously characterised as disease causing in another IPAH patient [7]. In this family, the missense mutation was present in the index patient and his uncle, who had been diagnosed with PAH aged 58 years. Two additional family members had already died of PAH. The grandfather had died of heart failure aged 49 years, while dyspnoea was unknown at the time of death.

A second HPAH family showed co-segregation of PAH with the heterozygous missense mutation c.413G > C p.(Arg138Pro) in the gene *SOX17.* Four family members of the youngest generation (III) had HPAH (Fig. 3B). Both female twins of the second generation had died before reaching the age of 30, however the cause of death was unclear. The twin II:5 died shortly post-partum while being an obligate carrier of the *SOX17* mutation. An additional sister of the index patient had died aged 13 months of an unknown cause. The only healthy sibling (III:6) of the index patient was a homozygous carrier of the *SOX17* wild-type allele (Fig. 3B). Since the father of the index patient (II:6) was also an obligate carrier a reduced penetrance has to be assumed. This is one of the largest *SOX17* families described up to date.

In the *KCNK3* family both children suffered from PAH (Fig. 3C) as described in detail in the next section.

### Two mutations in the same gene

Heritable PVOD, in contrast to PAH, is autosomal recessively inherited. In four PVOD patients bi-allelic variants in the gene *EIF2AK4* were identified (Table 3). Two were homozygous mutations while two were most likely compound heterozygotes. A co-segregation analysis to show the inheritance of each variant from a different parent has not been performed but the clinical diagnosis of the patients supported the presence of the pathogenic variants on two different alleles. One patient diagnosed with connective tissue disease APAH also carried two most likely bi-allelic pathogenic variants in the *EIF2AK4* gene (Table 3). The patient suffered from systemic lupus erythematosus, was treated with targeted PAH medication and had a reduced single-breath diffusion capacity of carbon monoxide (DLCO) of 15%.

Only in very rare cases homozygous variants have been identified in PAH patients so far. In this study, one young HPAH patient aged 5 years at diagnosis carried a homozygous 18 bp duplication in the gene *KCNK3* (Fig. 3C). The in-frame duplication was located in a repeat-free region of exon 1. Subsequently, the homozygous duplication was also identified in his affected little sister (Fig. 3C). She was diagnosed with PAH at three years of age and additionally presented with seizures. Both healthy parents were heterozygous carriers of the same duplication.

Two mutations were found in the same IPAH patient in the gene *TBX4* (Table 3). The first variant was a 56 bp insertion in exon 6 leading to a premature stop codon and thus, most likely resulted in nonsense mediated decay of the transcribed messenger RNA. The second variant was a missense mutation located in exon 8 of *TBX4* gene. Since no RNA was available from the patient, the respective messenger RNA could not be sequenced to identify whether both variants were present on the same allele (*in cis*) or on two different alleles (*in trans*).

Finally, two heterozygous *BMPR2* mutations were identified in the same IPAH patient. Both variants were located *in cis* in exon 8. The first variant was a missense mutation and the second variant was a deletion of 7 bp followed by the insertion of three base pairs leading to a frame shift and premature stop codon (see Additional file 1: Table S1). Due to the physical proximity of the two variants, their joined presence or absence could be verified in the 150 bp next generation sequencing reads, thus confirming their mono-allelic presence within the same inherited haplotype.

## Discussion

This study presents a large cohort of consecutive PAH patients who have been genetically assessed using most recent methods including all known PAH genes. Genetic defects were identified in 23% of the patients in a total of 11 PAH genes. This illustrates the benefit of the PAH-specific gene panel containing all known PAH genes. While 65% of mutations were identified in the main PAH gene *BMPR2,* 35% of disease-causing variants were located in ten other PAH genes. A genetic predisposition could be identified in six different forms of PAH. In three families co-segregating pathogenic variants in the genes *AQP1, KCNK3* and *SOX17* were revealed. Finally, bi-allelic variants were identified not only in *EIF2AK4*, but also in *KCNK3* and potentially *TBX4*.

### Value of PAH-specific panel diagnostics

The PAH-specific gene panel diagnostics developed in Heidelberg, Germany (European patent EP3507380) proved to be a valuable tool to identify genetic predisposition in PAH patients. The technique allows to sequence all known PAH genes in several patients at the same time [14]. Thus, it constitutes a time- and cost-efficient technique which can be continuously updated following the discovery of novel PAH genes. After starting with 13 PAH genes in 2017, the three genes *AQP1, ATP13A3* and *SOX17* were added after their description in 2018. In total, 24 pathogenic variants located in eight genes other than the traditionally Saner sequenced PAH/HHT genes *BMPR2, ACVRL1* and *ENG* were detected. As an alternative to panel sequencing whole exome or whole genome sequencing could have been applied. However, by focusing on genes with an established gene-disease relationship for PAH and by using our PAH-specific gene panel faster results could be obtained, with less extensive data and fewer variants of uncertain significance in genes likely not involved in the disease pathogenesis. The high coverage of at least × 100 for each base pair moreover guaranteed the detection of any potential mutations. In contrast, a continuous high coverage for whole exome sequencing is currently still hard to achieve. Thus, the PAH gene panel may be the method of choice for a targeted high-quality approach.

### Familial predisposition

The results of the PAH-specific gene panel are not only relevant for index patients but also for healthy family members. In particular *BMPR2* mutation carriers show a younger age of onset, worse haemodynamics and a faster disease progression [18] in comparison to patients without a predisposing variant. Thus, a closer supervision of these patients and potentially a faster therapy escalation should be considered. Family members, who currently show no symptoms of PAH but carry a familial mutation, should be regularly seen for a clinical screening at an expert center for pulmonary hypertension to identify a potential disease onset as early as possible [19, 20].

While in most HPAH families *BMPR2* is the causal gene, we showed that 23% of families carried mutations in six other PAH genes. This is the second study to identify a HPAH family with a pathogenic missense variant in the water channel gene *aquaporin 1* (*AQP1*). Three PAH families with mutations in *AQP1* have been published previously in a single study [7]. Of these families, two shared the familial missense variant, which was distinct from the one in our study. Thus, we describe the third familial pathogenic *AQP1* variant so far.

Mutations in the *SOX17* transcription factor gene are also very new findings in HPAH. At present, only two other HPAH families have been reported [7, 21]. Variants in this gene are increasingly identified in young PAH patients and patients with congenital heart disease APAH [13, 22]. While we observed a very early onset of PAH in three of four familial *SOX17* PAH patients with diagnosis at ages 1–3 years, no congenital heart disease was known.

In a third family, two children carried a homozygous duplication in the gene *KCNK3* and presented with a very early age of onset. Both heterozygous parents in our family were unaffected. In the only other previously described *KCNK3* family with a homozygous mutation PAH manifested in the homozygous 2 months old child and, in addition, post-partum in the heterozygous mother [23]. Apart from PAH, the sister of the index patient in our *KCNK3* family presented with seizures. For *KCNK3,* which encodes the two-pore potassium channel TASK1, this phenotype has never been described before. A pathogenetic link is likely as seizures are known to be caused by pathogenic variants in other potassium channels [24].

### Prevalence of mutations in PAH subgroups

The prevalence of disease-causing variants was the highest for HPAH patients, followed by drugs and toxins induced PAH, PVOD and IPAH patients. In these four groups we could identify mutations in 13–83% patients. In contrast, connective tissue disease APAH patients and congenital heart disease APAH patients both showed a 4% mutation prevalence. This frequency was in line with previous reports of around 3–5% [22]. Interestingly, while connective tissue disease can be a risk factor for PVOD development [25], characteristic bi-allelic variants in the *EIF2AK4* gene addition in a connective tissue disease APAH patient have only been described in two other patients before [22]. The congenital heart disease APAH patient carried a mutation in the *SOX17* gene. This is in concert with the data published by Zhu and colleagues who described a 3.2% mutation rate for *SOX17* in a cohort of congenital heart disease APAH patients [13]. No disease-causing variant could be identified in the two portal hypertension patients, two HIV-APAH patients nor the three patients with persistent PH of the newborn. However, all of these subgroups were too small to draw conclusions.

*BMPR2* mutation carriers presented with worse haemodynamics at diagnosis compared to patients without a genetic predisposition. Interestingly, this was not the case for patients with pathogenic variants in other genes than *BMPR2.* Nevertheless, not only *BMPR2*-muation carriers but also mutation carriers in the other genes received more often triple therapy at the time of this study than patients with no pathogenic variant. Whether this was due to the genetic variants leading to a more severe disease phenotype or the genetic diagnostic report prompted a faster combination therapy remains to be determined.

### Limitations

Patients in this study were analysed with the PAH-specific gene panel. While this targeted approach is the most cost and time effective method, there may be also pathogenic variants in other genes which could have been overlooked. However, since all established PAH genes were included on the panel, variants in other genes without a clear gene-disease relationship would have required further research and would have not been part of an official genetic diagnostic report. Similarly, variants which were located more than 20 base pairs into the introns were not analysed. For these variants extensive functional analyses would have been required to evaluate their potential effect on gene transcription. In addition, a detailed clinical characterisation of mutation carriers in genes other than *BMPR2* would have been interesting for each gene. However, the respective numbers were too low to permit such analysis.

## Conclusions

Of the 325 consecutive PAH patients referred for genetic testing in this study approximately each fifth patient had a genetic background leading to PAH. Pathogenic variants were not only identified in the *BMPR2* gene but also in ten further PAH genes. Genetic predisposition was not restricted to HPAH, IPAH and PVOD but could also be identified in some patients with APAH. HPAH families with mutations in the genes *AQP1, SOX17* or *KCNK3* underline the broad spectrum of mutations. The PAH-specific gene panel based on next generation sequencing proofed to be readily extendable for newly discovered genes to include all known PAH genes at any time point. Hence, our approach provided a valuable, timely, high-quality tool for the clinical diagnostic work-up of PAH patients.

## Supplementary Information

**Additional file 1: Table S1.** (Likely) pathogenic BMPR2 variants identified in the cohort.

## Data Availability

The dataset supporting the conclusions of this article is included within the article (and its additional file).

## Abbreviations

ACMG: American College of Medical Genetics and Genomics
*ACVRL1*: Activin receptor like kinase 1
APAH: Associated pulmonary arterial hypertension
*AQP1*: Aquaporin 1
*BMPR2*: Bone morphogenetic protein receptor 2
DLCO: Diffusion capacity of carbon monoxide
*ENG*: Endoglin
HPAH: Heritable pulmonary arterial hypertension
HIV: Human immunodeficiency virus
IPAH: Idiopathic pulmonary arterial hypertension
MLPA: Multiplex ligation-dependent probe amplification
NGS: Next generation sequencing
PAH: Pulmonary arterial hypertension
PVOD: Pulmonary veno-occlusive disease
TGF-β: Transforming growth factor beta
