# Supplementary material for: Gene panel diagnostics reveals new pathogenic variants in pulmonary arterial hypertension
Source: Respir Res. 2022 Mar 27;23:74. doi: 10.1186/s12931-022-01987-x (PMC8962083; doi:10.1186/s12931-022-01987-x)
Supplement: Supplementary file 1 — Additional file 1: Table S1. (Likely) pathogenic BMPR2 variants identified in the cohort. [file 12931_2022_1987_MOESM1_ESM.docx]

**Supplementary Table S1. (Likely) pathogenic *BMPR2* variants identified in the cohort**

| **Gene** | **Exon** | **DNA** | **Protein** | **CADD score** | **GnomAD count** | **Diagnosis** |
| --- | --- | --- | --- | --- | --- | --- |
| *BMPR2* | 1 | c.1-?_c.76+?del | Exon 1 deletion | NA (Deletion) | 0 | 2 IPAH, 1 drugs and toxins induced PAH |
| *BMPR2* | 1 | c.26_41delGGCGGGTGCCCTGGCT | p.(Trp9Tyrfs*33) | NA (Deletion) | 0 | IPAH |
| *BMPR2* | 1 | c.27delG | p.(Trp9Cysfs*38) | NA (Deletion) | 0 | IPAH |
| *BMPR2* | 1 | c.39G>A | p.(Trp13*) | NA (Nonsense) | 0 | HPAH |
| *BMPR2* | 1 | c.45delA | p.(Trp16Glyfs*31) | NA (Deletion) | 0 | IPAH |
| *BMPR2* | 1 | c.48G>A | p.(Trp16*) | NA (Nonsense) | 0 | IPAH |
| *BMPR2* | 1 | c.49delA | p.(Thr17Profs*30) | NA (Deletion) | 0 | HPAH |
| *BMPR2* | 1 | c.76+1G>T | (splice defect) | 34 | 0 | HPAH |
| *BMPR2* | 1 | c.76+5G>A | intronic | 24 | 0 | HPAH |
| *BMPR2* | 2 | c.247+5G>A | RNA: r.141_247del; r.189_247del | NA (Splice site loss) | 0 | HPAH |
| *BMPR2* | 2-3 | c.77-?_c.418+?del | Exon 2-3 deletion | NA (Deletion) | 0 | 2 HPAH (unrelated) |
| *BMPR2* | 3 | c.287_288delAT | p.(Tyr96*) | NA (Deletion) | 0 | HPAH |
| *BMPR2* | 3 | c.341G>C | p.(Arg114Pro) | 29 | 0 | IPAH |
| *BMPR2* | 3 | c.346T>C | p.(Cys116Arg) | 26 | 0 | HPAH |
| *BMPR2* | 3 | c.377A>C | p.(Asn126Thr) | 26 | 0 | 2 HPAH (unrelated) |
| *BMPR2* | 3 | c.417_418+2delCAGT | p.(Pro141Thrfs*5) | NA (Deletion) | 0 | HPAH |
| *BMPR2* | 3 | c.418+2T>G | intronic | 35 | 0 | HPAH |
| *BMPR2* | 4 | c.439C>T | p.(Arg147*) | Nonsense | 1 | IPAH |
| *BMPR2* | 5 | Alu-element insertion | RNA: r.622_852del; r.622_714del | NA (Deletion) | 0 | HPAH [1] |
| *BMPR2* | 6 | c.631C>T | p.(Arg211*) | Nonsense | 0 | 3 HPAH (unrelated) |
| *BMPR2* | 6 | c.689_690delAA | p.(Lys230Serfs*25) | NA (Deletion) | 0 | 2 HPAH (unrelated) |
| *BMPR2* | 6 | c.731delA | p.(Lys244Argfs*8) | NA (Deletion) | 0 | HPAH |
| *BMPR2* | 6 | c.823dupT | p.(Tyr275Leufs*23) | NA (Deletion) | 0 | IPAH |
| *BMPR2* | 6-9 | c.622-?_c.1276+?del | Exon 6-9 deletion | NA (Deletion) | 0 | IPAH |
| *BMPR2* | 7 | c.853-2A>G | intronic | 34 | 1 | IPAH |
| *BMPR2* | 8 | c.1041T>G | p.(Cys347Trp) ^a^ | 26 | 0 | IPAH |
| *BMPR2* | 8 | c.1044_1050delinsGGT | p.(Ile349Thrfs*6) ^a^ | NA (Indel) | 0 | IPAH |
| *BMPR2* | 8-10 | c.968-?_c.1413+?dup | Exon 8-10 duplication | Duplication | 0 | HPAH |
| *BMPR2* | 10 | c.1277-2A>G | intronic | 33 | 0 | IPAH |
| *BMPR2* | 11 | c.1414-?_c.1586+?del | Exon 11 deletion | NA (Deletion) | 0 | IPAH |
| *BMPR2* | 11 | c.1414-?_c.1586+?dup | Exon 11 duplication | NA (Duplication) | 0 | HPAH |
| *BMPR2* | 11 | c.1468_1469dupGC | p.(Arg491Leufs*16) | NA (Duplication) | 0 | HPAH |
| *BMPR2* | 11 | c.1471C>T | p.(Arg491Trp) | 32 | 0 | 2 IPAH, 2 HPAH (unrelated) |
| *BMPR2* | 11 | c.1472G>A | p.(Arg491Gln) | 32 | 0 | HPAH |
| *BMPR2* | 12 | c.2009dupC | p.(Glu672Argfs*3) | NA (Duplication) | 0 | IPAH |
| *BMPR2* | 12 | c.2342T>A | p.(Leu781*) | NA (Nonsense) | 0 | IPAH |
| *BMPR2* | 12 | c.2347_2348insTC | p.(Gln783Leufs*10) | NA (Insertion) | 0 | HPAH |
| *BMPR2* | 12 | c.2413dupA | p.(Thr805Asnfs*8) | NA (Duplication) | 0 | HPAH |
| *BMPR2* | 12 | c.2421delT | p.(Asn807Lysfs*32) | NA (Deletion) | 0 | HPAH |
| *BMPR2* | 12 | c.2530C>T | p.(Gln844*) | Nonsense | 0 | HPAH |
| *BMPR2* | 12 | c.2695C>T | p.(Arg899*) | Nonsense | 0 | IPAH |

^a^ together with 2^nd^ variant in same gene; CADD score: Combined Annotation Dependent Depletion score, summation score based on different *in* *silico* prediction programmes; GnomAD: Genome Aggregation Consortium Database with n=125,748 samples; HPAH: heritable pulmonary arterial hypertension; IPAH: idiopathic pulmonary arterial hypertension. Used Ensembl transcript and reference sequence for the *BMPR2* gene: ENST00000374580.8, NM_001204.

**Supplementary Reference:**

22 Hinderhofer K, Fischer C, Pfarr N, Szamalek-Hoegel J, Lichtblau M, Nagel C, Egenlauf B, Ehlken N, and Grünig E. Identification of a new intronic *BMPR2*-mutation and early diagnosis of heritable pulmonary arterial hypertension in a large family with mean clinical follow-up of 12 years. *PLoS One* **2014,** 9:e91374.

1. Hinderhofer K, Fischer C, Pfarr N, Szamalek-Hoegel J, Lichtblau M, Nagel C, Egenlauf B, Ehlken N, Grünig E: Identification of a new intronic *BMPR2*-mutation and early diagnosis of heritable pulmonary arterial hypertension in a large family with mean clinical follow-up of 12 years. PLoS One. 2014; 9:e91374.
